# Monoclonal Antibody Preparation and Epitope Identification for *Brucella melitensis* Elongation Factor Tu

**DOI:** 10.3389/fmicb.2019.01878

**Published:** 2019-08-13

**Authors:** Ningning Zhao, Yue Jiang, Shuzhen Ming, Sidang Liu, Xiaomin Zhao, Fangkun Wang

**Affiliations:** ^1^Department of Preventive Veterinary Medicine, College of Veterinary Medicine, Shandong Agricultural University, Tai’an, China; ^2^Shandong Provincial Key Laboratory of Animal Biotechnology and Disease Control and Prevention, Shandong Agricultural University, Tai’an, China; ^3^Shandong Provincial Engineering Technology Research Center of Animal Disease Control and Prevention, Shandong Agricultural University, Tai’an, China

**Keywords:** monoclonal antibody, epitope, EF-Tu, affinity tag, *Brucella melitensis*

## Abstract

Elongation factor thermo-unstable (EF-Tu), an abundant multifunctional protein, is pivotal during protein synthesis and is an important antigen. Few studies have addressed the role of this protein in *Brucella* species, and the epitopes of this protein have not been reported. Here, we describe a monoclonal antibody (McAb), BD_6_, for EF-Tu in *Brucella melitensis*. Using western blotting involving a series of partially overlapping recombinant EF-Tu truncation peptides, a novel linear B-cell epitope, ^110^QTREHIL^116^ (EF), was identified. Alanine-scanning mutagenesis revealed that residues Q^110^, T^111^, R^112^, I^115^, and L^116^ were core residues involved in recognition. Sequence alignment suggested that the epitope peptide was conserved among bacterial species but differed by one amino acid residue (I^115^) from the host sequence. The epitope peptide was recognized by sera from *B. melitensis*-infected mice, and while recombinant epitope peptide induced a strong humoral immune response, the corresponding mouse peptide, QTREHLL, did not. These results suggested that I^115^ may be the key residue for the host immune system to distinguish between bacterial and self epitope EF sequences. Indirect immunofluorescence and western blotting assays showed that epitope peptide could be used in *Saccharomyces cerevisiae*, human embryonic kidney cell (HEK-293), and chicken fibroblast cell (DF1) expression systems and immunoprecipitation assay. Together, our results suggested that the McAb BD_6_ is a useful tool for further investigation of the potential functions of the EF-Tu protein in pathogen-host interactions, and that the epitope tag may be useful for application as a novel affinity tag to identify other bacterial pathogens, especially convenient for the identification of intracellular bacteria.

## Introduction

The protein elongation factor thermo-unstable (EF-Tu) is abundant both in prokaryotic and eukaryotic cells, reaching concentrations almost ten times higher than that of ribosomes under rapid growth conditions (Furano, 1975). EF-Tu is a cytoplasmic protein and plays a central role in protein synthesis by mediating the transport of aminoacyl-tRNA to the codon recognition site of ribosomes (Kaziro, 1978). However, recent studies have suggested that the EF-Tu proteins of several prokaryotic plant and animal pathogens are exposed on the cell surface (Balasubramanian et al., 2008; Barel et al., 2008; Wolff et al., 2013; Amimanan et al., 2017) and that surface-located EF-Tu proteins play a pivotal role in bacterial adhesion, invasion, and host immune evasion (Jonak, 2007; Kunert et al., 2007; Mohan et al., 2014; Amimanan et al., 2017). *Brucella melitensis* is an important zoonotic pathogen. In Yang et al. (2011), identified *B. melitensis* EF-Tu as a candidate antigen using an immunoproteomics approach. Wang et al. (2013) then found that EF-Tu-encoding gene *tuf2* plays an important role in the virulence attenuation of *B. melitensis* vaccine strain M5-90. The specific virulence mechanism and other functions of the EF-Tu protein remain unclear, and molecular tools needed to carry out an analysis, including a monoclonal antibody (McAb) targeting the *Brucella* EF-Tu protein, have yet to be developed.

McAbs were first reported in 1975 (Kohler and Milstein, 1975) and have revolutionized many areas of vaccine development and protein discovery. For example, McAbs are widely used in disease diagnosis, functional protein studies, and in monoclonal antibody-based therapies, among many other applications (Kivi et al., 2016). Epitope mapping using McAbs provides a platform for studying antigen structure and developing epitope vaccines (Tissot et al., 2010). Together with their corresponding epitopes, McAbs also have wide-ranging experimental applications, including western blot analysis, enzyme-linked immunosorbent assays (ELISA), co-immunoprecipitation, endogenous localization, and affinity purification (Kimple et al., 2013; Liu et al., 2016). In the present study, we generated an anti-*B. melitensis* EF-Tu mouse McAb, identified its linear B-cell epitope, and examined the antigenicity of the epitope peptide. To the best of our knowledge, our findings mark the first B-cell linear epitope in the EF-Tu protein from *B. melitensis* and provide a tool for studying the function and mechanism of *B. melitensis* EF-Tu.

## Materials and Methods

### Ethics Statement

All the experiments were conducted at biosafety level 1 laboratories, strictly in accordance with laboratory biosafety regulations. The study protocol and all animal experiments were approved by the Animal Care and Use Committee of Shandong Agricultural University, Tai’an, China (SCUC permission no. SDAUA-2015-015), and were performed in strict accordance with experimental animal regulation ordinances defined by China National Science and Technology Commission. The female BALB/c mice were housed in a suitable habitat, without psychological trauma or unnecessary pain throughout the study. Mice were housed in 800-cm^2^ plastic cages (five mice per cage) under a normal light-dark cycle and provided with standard laboratory food and water *ad libitum*. All mice were euthanized with an overdose of isoflurane (5%). Exposure to isoflurane (5%) was continued for at least 1 min after breathing had ceased.

### Strains, Cell Lines, and Experimental Animals

The vectors pET30a-EF-Tu (from our own collection) and pGEX-6p-1 (TaKaRa, Dalian, China) were used to obtain recombinant His-tagged and glutathione S-transferase (GST)-tagged *B. melitensis* EF-Tu proteins, respectively. *Escherichia coli* Top10 cells (Invitrogen, Carlsbad, CA, United States) were used for all cloning experiments, while *E. coli* BL21(DE3) (Invitrogen) was used for the protein expression assay. Anti-EtMIC2 (microneme 2 protein from the protozoan parasite *Eimeria tenella*) McAbs were prepared in our laboratory as described previously (Liu et al., 2014). Mouse serum infected with *B. melitensis* artificially was kindly provided by Prof. Bu, Harbin Veterinary Research Institute. SP20 mouse myeloma cells were purchased from the American Type Culture Collection (Manassas, VA, United States) and cultured in Gibco Dulbecco’s modified Eagle’s medium (Thermo Fisher Scientific, Waltham, MA, United States) supplemented with 10% (w/v) fetal bovine serum (FBS; Thermo Fisher Scientific). Female BALB/c mice aged 4–5 weeks were purchased from Vital River Laboratories (Beijing, China).

### Preparation of Recombinant Proteins

*Brucella melitensis* EF-Tu-encoding gene *tuf2* was amplified from pET30a-EF-Tu using primers BM-GST-F (5′-TTG GATCCATGGCAAAGAGTAAGTTTGAAC-3′) and BM-GST-R (5′-TTCTCGAGTTACTCGATGATCGAC-3′) and cloned into vector pGEX-6p-1, generating recombinant plasmid pGEX-EF-Tu. pGEX-EF-Tu and pET30a-EF-Tu were then separately transformed into electrocompetent *E. coli* BL21(DE3) cells. Following induction with isopropyl-β-D-thiogalactopyranoside, recombinant proteins were purified using a His-Bind Purification Kit (TransGen Biotech, Beijing, China) or a ProteinIso GST Resin Kit (TransGen Biotech) and confirmed by sodium dodecyl sulfate-polyacrylamide gel electrophoresis (SDS–PAGE) analysis. Protein concentrations were determined using a Bio-Rad Protein Assay Kit (Bio-Rad Laboratories, Hercules, CA, United States) as per the manufacturer’s instructions.

### Generation of Hybridoma Cells

Hybridoma cells were produced as per the method described by Liu et al. (2014). Briefly, mice were immunized subcutaneously with purified His-EF-Tu protein (50 μg/mouse) suspended in Freund’s complete adjuvant (FCA; Sigma-Aldrich, St. Louis, MI, United States). Following two additional immunizations with the same dose of Freund’s incomplete adjuvant (FIA; Sigma, United States) at 2-week intervals, immunoreactivity against EF-Tu was validated and a final booster immunization of purified His-EF-Tu protein (100 μg/mouse) was given without adjuvant. Four days after booster immunization, mice were euthanized, their spleens removed, and spleen cells isolated using standard techniques. The spleen cells were fused with SP20 myeloma cells to generate hybridomas as described by Hadji-Ghasemi et al. (2003). The immunoglobulin subclass was determined using an IsoStrip Mouse Monoclonal Antibody Isotyping Kit (Sigma-Aldrich) as per the manufacturer’s instructions. Selected cell clones were then cultured in the peritoneal cavities of BALB/c mice to obtain ascetic fluid. McAbs were then purified from the resulting ascites fluid using a Protein G Spin Purification Kit (Pierce Biotechnology, Rockford, IL, United States) and stored at −80°C until further use. The resulting anti-*B. melitensis* EF-Tu McAb was named BD_6_.

### Immunofluorescent Staining of Yeast Surface-Displayed EF-Tu

Yeast surface display of *B. melitensis* EF-Tu protein was used to determine the specificity of BD_6_ as per the method described by Sun et al. (2014b). Briefly, *B. melitensis* EF-Tu-encoding gene *tuf2* was cloned into pCTCON2 using the primers described above. The recombinant plasmid was transformed into *Saccharomyces cerevisiae* cells for the display of *B. melitensis* EF-Tu on the cell surface. Resulting yeast cells displaying EF-Tu were incubated with BD_6_ (1:1,000) as the primary antibody, with fluorescein isothiocyanate (FITC)-conjugated goat anti-mouse IgG [H + L] (Cowin Biotech, Beijing, China) then used as secondary antibody. After three washes with PBS, the yeast cells were resuspended in PBS and observed under a fluorescence microscope (TE-2000-S; Nikon, Tokyo, Japan). Yeast cells labeled with anti-EtMIC2 McAb acted as controls for non-specific antibody binding. *S. cerevisiae* transformed with empty plasmid was stained in the same way and used as a negative control.

### Identification of the Position of the Linear Epitope

To map the linear epitope, a GST-tagged fragment of the *B. melitensis* EF-Tu gene was designed and expressed in *E. coli* BL21(DE3). Three overlapping fragments of the gene were amplified using specific primers (Supplementary Table 1) and cloned into vector pGEX-6p-1, generating recombinant plasmids pGEX-EF-Tu-1-1, pGEX-EF-Tu-1-2, and pGEX-EF-Tu-1-3. The recombinant proteins were expressed and purified as described above and then analyzed by western blotting with BD_6_ as primary antibody and horseradish peroxidase (HRP)-conjugated goat anti-mouse IgG (Cowin Biotech) as secondary antibody.

Based on the results of the western blot, two fragments (EF-Tu-2-1 and EF-Tu-2-2) of the positive region were amplified (Supplementary Table 1), cloned, and expressed as described above to determine the position of the epitope. Recombinant proteins were detected by western blotting using BD_6_ as described above. A further five fragments (EF-Tu-3-1, EF-Tu-3-2, EF-Tu-3-3, EF-Tu-3-4, and EF-Tu-3-5) were then amplified (Supplementary Table 1), expressed, and examined in the same way. Lastly, four fragments (EF-Tu-4-1, EF-Tu-4-2, EF-Tu-4-3, and EF-Tu-4-4) were designed by reducing the number of amino acids one by one on each side until the accurate position of the epitope was determined (Supplementary Table 1). The epitope was referred to as EF.

### Alanine-Scanning Mutagenesis

The key amino acid residues for antigen binding to the McAb were identified by western blotting and yeast expression. For western blotting, a series of GST-fusion mutant peptides where individual residues of the epitope peptide were substituted with alanine were designed (Supplementary Table 2). The mutant peptides were then expressed and analyzed by western blotting using anti-EF-Tu McAb as the primary antibody.

Overlap PCR was used to generate the mutant peptides. Briefly, to generate a Q110A substitution in the EF-Tu-encoding gene, two overlapping gene fragments were generated by PCR using primers P-EF-Tu-F1/P-EF-Tu-(110)-R1 and P-EF-Tu-(110)-R2/P-EF-Tu-R1 (Supplementary Table 3), respectively. The mutant gene was then generated by overlap PCR using the previous PCR product as template and P-EF-Tu-F1/P-EF-Tu-R1 as primers. The remaining mutant EF-Tu genes containing single amino acid substitutions were obtained in the same way using primers listed in Supplementary Table 3. The mutant EF-Tu proteins were then displayed on the surface of *S. cerevisiae* cells as described above. Following incubation with BD_6_ as the primary antibody and FITC-conjugated goat anti-mouse IgG [H + L] as the secondary antibody, yeast cells with bound antibody were detected by fluorescence microscopy and using a BD LSRFortessa cell analyzer (BD Biosciences, Franklin Lakes, NJ, United States).

### Analysis of Biological Information

The spatial distribution of the identified epitope in the *B. melitensis* EF-Tu protein was analyzed by mapping the epitope locations on a 3-D model using the SWISS-MODEL online server. The antigenic index of EF-Tu was analyzed using PROTEIN software (DNASTAR Inc., Madison, WI, United States).

### Mouse Immunization Assays

Glutathione S-transferase-tagged *B. melitensis* and mouse recombinant EF proteins were obtained as described above. To assess the level of the EF fusion proteins to induce humoral immunity, 20 female BALB/c mice were divided into four groups (*n* = 5 mice per group). At 4 weeks of age, the mice were immunized subcutaneously with 50 μg of GST-EF (*B. melitensis*), GST-EF (Mouse), or purified GST protein, or with 100 μL of PBS, in FCA. At 2 weeks post-immunization, the mice were given a booster immunization using the same dose of protein in FIA. On the day of the booster immunization and at 7 days post-booster immunization, the levels of anti-EF (*B. melitensis*/mouse) synthetic peptide serum antibodies were estimated by enzyme-linked immunosorbent assay (ELISA).

### Dot Blot Assays

Mouse serum samples infected with *B. melitensis* were detected by dot blot as described by Sun et al. (2019). Briefly, approximately 500 ng of synthetic peptide was spotted onto nitrocellulose membranes (Millipore, Bedford, MA, United States) in the center of the grid. After blocking with 5% (w/v) bovine serum albumin in TBST buffer, membranes were incubated with serum samples or anti-EF-Tu McAb (positive control) as the primary antibody for 1 h at 37°C. The membranes were then washed three times with TBST buffer and incubated with HRP-conjugated goat anti-bovine IgG (H + L; Sigma-Aldrich) for 1 h at 37°C. After washing, enhanced chemiluminescence reagent was used to visualize the reaction.

### Expression of Heterogenous Genes Fused With an EF Tag

To examine the effects of an epitope EF tag in a yeast expression system, EtMIC2 was used as a foreign gene to be displayed in yeast cells. Epitope EF tag was fused to the C-terminus of EtMIC2 in vector pCTCON2-EtMIC2 (Sun et al., 2014a). Yeast cells were then transfected with pCTCON2-EtMIC2 or pCTCON2-EtMIC2-EF and detected by indirect immunofluorescence using anti-EtMIC2 McAb. The expression of EtMIC2-EF and EtMIC2 on yeast cells was then quantified by flow cytometry as described by Sun et al. (2014a).

To examine the effects of a BD_6_ epitope tag in mammalian and avian expression systems, an epitope EF tag was fused to the C-terminus of EGFP in vector pEGFP-C1. The recombinant plasmid was transfected into human embryonic kidney cells (HEK-293) and chicken fibroblasts (DF1) as described previously (Liu et al., 2016). At 24 h post-transfection, cells were observed by fluorescence microscope, while cell lysates were collected as described previously (Liu et al., 2016) and analyzed by western blotting.

### Immunoprecipitation (IP) of Transiently Expressed EGFP-BD_6_ Epitope

To examine the specificity of BD_6_ for immunoprecipitation, 500 μL of protein extract from EGFP-EF-expressing HEK-293 cells was mixed with 2 μg of BD_6_ and incubated for 2 h at 4°C. A 25-μL volume of Protein G Sepharose (GE Healthcare Life Sciences, Pittsburgh, PA, United States) was added to the supernatant and incubated for 2 h at 4°C. The supernatant was then discarded after centrifugation and the Sepharose was washed three times. Proteins were analyzed by western blotting using anti-GFP McAb as the primary antibody.

## Results

### Generation and Identification of Anti-EF-Tu McAb BD_6_

**FIGURE 1:**
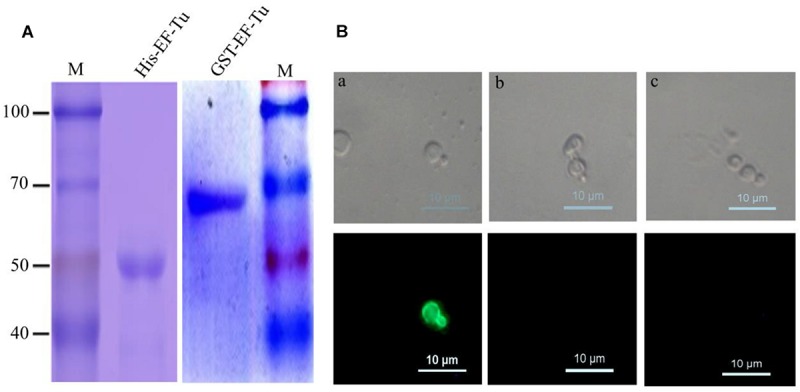
Identified McAbs that recognize EF-Tu protein. **(A)** SDS–PAGE analysis of purified His-Tag and GST-Tag *Brucella melitensis* EF-Tu protein. **(B)** Analysis of specificity of McAb BD_6_ by yeast surface displays. **(a)** Yeast cells displaying *B. melitensis* EF-Tu protein were immunofluorescently labeled with McAb BD_6_; **(b)** yeast cells with empty plasmid were immunofluorescently labeled with McAb BD_6_; **(c)** yeast cells displaying *B. melitensis* EF-Tu protein with anti-EtMIC2 protein McAb as a non-specific binding McAb control.

To obtain anti-EF-Tu McAbs, His-tagged and GST-tagged EF-Tu proteins were expressed and purified. SDS–PAGE analyses revealed that the two recombinant proteins were successfully purified and had the correct molecular mass (Figure 1A). After fusion and screening, a hybridoma cell line stably expressing and secreting antibodies against GST-EF-Tu was obtained. The resulting McAbs were designated BD_6_. Subclass detection showed that BD_6_ belonged to the IgG1 subclass of antibodies that express a κ light chain.

Because naturally expressed protein is the best substrate for identifying McAbs (Liu et al., 2014; Chen et al., 2018), the yeast surface display technique was used to test the specificity of BD_6_. Indirect immunofluorescence assays showed strong immunofluorescence on the surface of yeast cells expressing *B. melitensis* EF-Tu (Figure 1Ba) but not on those transformed with the empty vector (Figure 1Bb). Additionally, no fluorescence was observed for *B. melitensis* EF-Tu-expressing yeast cells labeled with non-specific antibody (Figure 1Bc). The cell line specifically secreting McAb BD_6_ has been stored at the China General Microbiological Culture Collection Center (no. 10952).

### Identification of the Epitope Recognized by BD_6_

**FIGURE 2:**
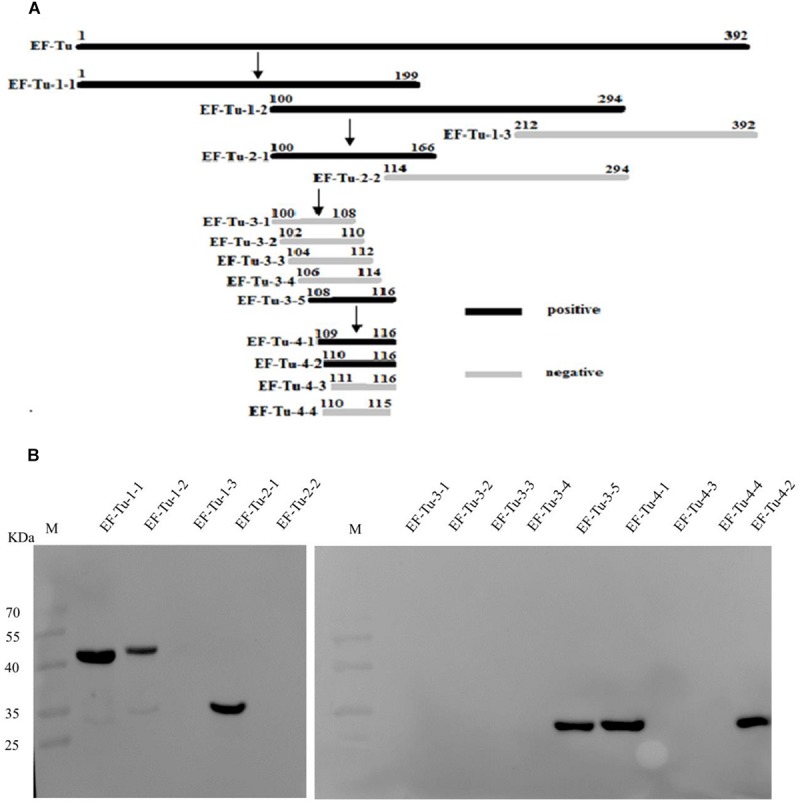
Identification of epitope recognized by McAb BD_6_. **(A)** Summary design of overlapping fragments of EF-Tu, with accurate position of epitope recognized by McAb BD_6_ deduced at ^110^Gln Thr Arg Glu His Ile leu^116^ in *B. melitensis* EF-Tu protein. **(B)** Analysis of various overlapping fusion proteins of EF-Tu expressed in *E. coli* BL21 (DE3) by western blotting using McAb BD_6_ as primary antibody.

To accurately map the position of the BD_6_ epitope, 10 overlapping fragments of the EF-Tu-encoding gene were amplified (Figure 2A). The corresponding amino acid sequences are shown in Supplementary Table 4. Following expression in *E. coli* BL21(DE3), western blotting showed that BD_6_ reacted with fragments EF-Tu-1-1, EF-Tu-1-2, EF-Tu-2-1, and EF-Tu-3-5, but not with EF-Tu-1-3, EF-Tu-2-2, EF-Tu-3-1, EF-Tu-3-2, EF-Tu-3-3, or EF-Tu-3-4 (Figure 2B). Four additional fragments were designed to more specifically determine the position of the epitope by decreasing the length of the peptide by one amino acid at a time from both sides of the sequence (Figure 2A). Western blotting showed that BD_6_ reacted with EF-Tu-4-1 and EF-Tu-4-2 but not with EF-Tu-4-3 or EF-Tu-4-4 (Figure 2B). Based on the results of western blotting, the epitope within *B. melitensis* EF-Tu that is recognized by BD_6_ was deduced to be ^110^QTREHIL^116^.

### Critical Residues Responsible for the Activity of BD_6_

**FIGURE 3:**
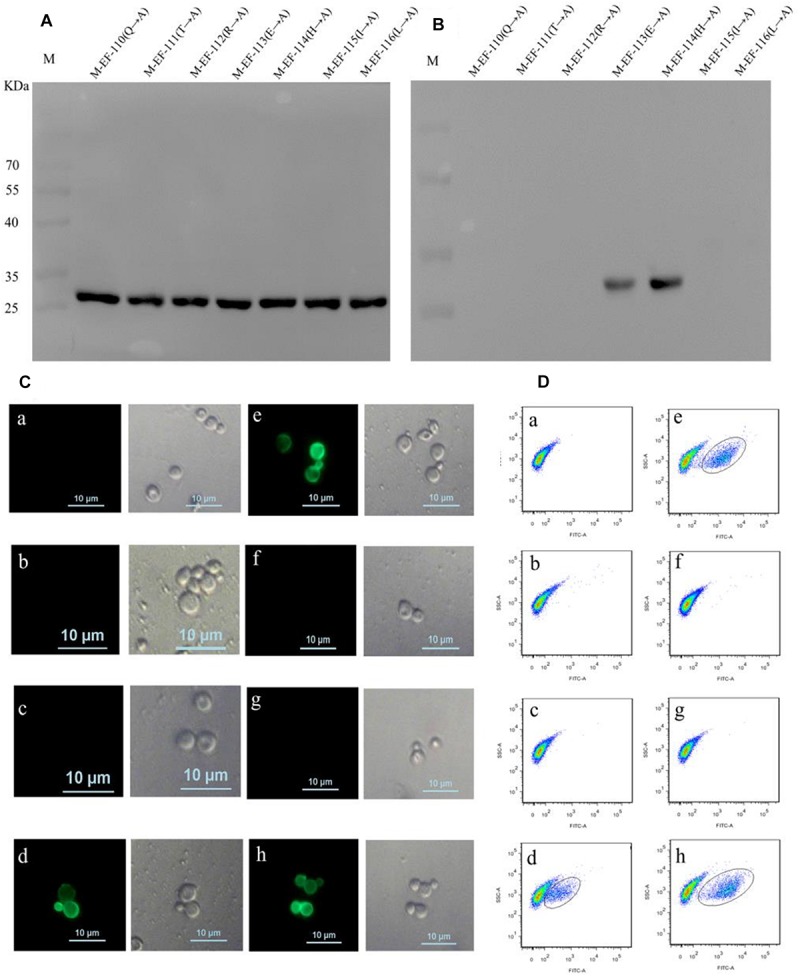
Identification of the crucial residue of the epitope to McAb BD_6_. **(A)** Western blotting analysis of seven mutagenesis epitope peptides expressed in *E. coli* BL21 (DE3) using anti-GST McAb as primary antibody. **(B)** Western blotting analysis of seven mutagenesis epitope peptides by western blotting to confirmed central sequence of epitope, using McAb BD_6_ as primary antibody. **(C,D)** Indirect immunofluorescence assay **(C)** and flow cytometry **(D)** analysis yeast cells displayed seven mutagenesis EF-Tu proteins. **(a)** Yeast cell displaying P-EF-Tu-(Q^110^-A) protein; **(b)** yeast cell displaying P-EF-Tu-(T^111^-A) protein; **(c)** yeast cell displaying P-EF-Tu-(R^112^-A) protein; **(d)** yeast cell displaying P-EF-Tu-(E^113^-A) protein; **(e)** yeast cell displaying P-EF-Tu-(H^114^-A) protein; **(f)** yeast cell displaying P-EF-Tu-(I^115^-A) protein; **(g)** yeast cell displaying P-EF-Tu-(L^116^-A) protein; and **(h)** yeast cell displaying *B. melitensis* EF-Tu protein as positive control. BD_6_ as primary antibody and fluorescein isothiocyanate (FITC)-conjugated goat anti-mouse IgG [H + L] as secondary antibody.

To define the key residues contributing to the activity of epitope EF, seven mutant epitope peptides with GST tags were designed and separately expressed in BL21 before being screened by immunoblotting using anti-GST McAb as the primary antibody (Figure 3A). Western blotting showed that BD_6_ reacted with M-EF-113 and M-EF-114 but not with M-EF-110, M-EF-111, M-EF-112, M-EF-115, or M-EF-116 (Figure 3B).

To further confirm the key amino acid residues, seven mutant EF-Tu gene constructs containing alanine substitutions at single amino acid residues were generated by overlap PCR. The resulting EF-Tu proteins were individually displayed on the surface of yeast cells. Binding of BD_6_ to the mutant EF-Tu proteins on the yeast cell surfaces was then detected by fluorescence microscopy and using a cell analyzer. Indirect immunofluorescence assays showed strong immunofluorescence on the surface of yeast cells displaying P-EF-Tu-(E113A), P-EF-Tu-(H114A), and wild-type EF-Tu protein (Figures 3Cd,e,h). No immunofluorescence was detected for yeast cells displaying the remaining mutant EF-Tu proteins. Flow cytometry confirmed that BD_6_ recognized P-EF-Tu-(E113A), P-EF-Tu-(H114A), and wild-type EF-Tu protein, but did not bind to the other mutant proteins (Figure 3D). These results indicated that residues Q^110^, T^111^, R^112^, I^115^, and L^116^ were critical for binding of BD_6_ to the EF epitope. In contrast, substitutions at E^113^ and H^114^ had no effect on the binding of the McAb to the epitope.

### Conservation of the Epitope Sequence Recognized by BD_6_

**FIGURE 4:**
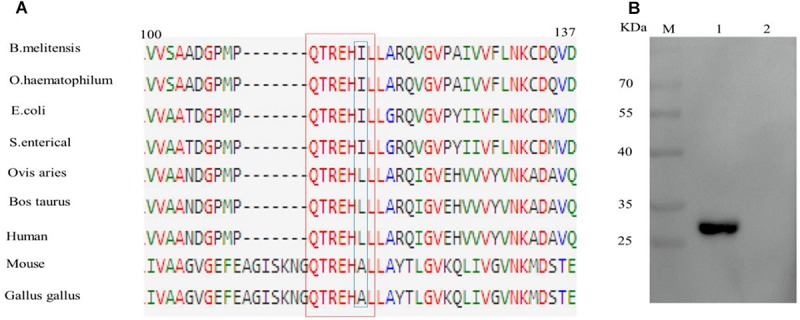
Analysis of the difference of epitope amino acids among different species. **(A)** Alignment of EF-Tu protein amino acid sequences surrounding the epitope region. The BD_6_ epitope amino acid residues are in red box. **(B)** Western blotting analysis of the reactivity between BD_6_ and QTREHLL (human). 1, GST-QTREHIL (*B. melitensis*) recombinant protein, positive control; 2, GST-QTREHLL (human) recombinant protein.

Sequence alignment of epitope motifs from different bacterial species found that the epitope sequence QTREHIL is highly conserved amongst a range of bacterial species (Figure 4A). However, a single amino acid (I^115^) difference was observed between bacterial and host epitope sequences (Figure 4A, blue box). The reactivity between BD_6_ and the human EF epitope (QTREHLL) was then analyzed. Western blotting showed that BD_6_ did not recognize the human peptide sequence (Figure 4B).

### Antigenicity of Epitope EF

**FIGURE 5:**
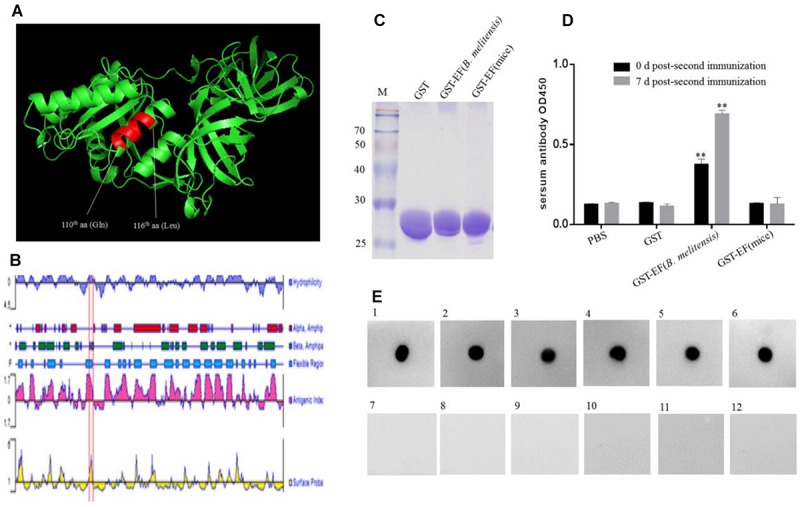
Identification of the immunogenicity of epitope EF. **(A)** Localization of the identified BD_6_ epitope in a partially predicted 3-D structure of *B. melitensis* EF-Tu protein is highlighted in an α-helix. **(B)** The antigenic index of EF-Tu protein was predicted by DNAstart and the epitope ^110^QTREHIL^116^ was located on a highlighted antigenic index region (red box). **(C)** SDS–PAGE analysis of purified GST, GST-EF (*B. melitensis*), GST-EF (mice) protein. **(D)** The level of serum antibody IgG immunized with GST-EF (*B. melitensis*), GST-EF (mice) protein was detected by ELISA on 0 day and 7 days post-second immunization. ^∗∗^*p* < 0.001 represents the significant difference compared with PBS group. **(E)** Dot-blot analysis of *B. melitensis*-infected mouse sera with synthetic epitope EF peptide as antigen. 1–5, *B. melitensis*-infected sera; 7–11, no-*B. melitensis*-infected sera; 6, BD_6_ McAb as positive control; 12, Membrane incubated without primary antibody as control.

The 3-D structure of *B. melitensis* EF-Tu was predicted using the SWISS-MODEL online server (Figure 5A). The template is ancient sequence-reconstructed Elongation Factor Tu (SMTL ID: 6gfu. 1), with 99% query coverage and 86.89% identity. Structural analysis showed that the epitope formed part of an α-helix and was located in a region with a high antigenic index and high hydrophilicity (Figure 5B). These findings suggesting that epitope EF may be a vital B-cell epitope within the EF-Tu protein of *B. melitensis*. To confirm the antigenicity of epitope EF, mice were immunized with purified *B. melitensis* or mouse GST-tagged EF proteins (Figure 5C). Serum antibodies induced by the fusion proteins were then detected by ELISA (Figure 5D). Results showed significantly higher production of serum antibodies against the *B. melitensis* GST-EF epitope peptide compared with GST alone at both 0 and 7 days post-booster immunization (Figure 5D). There was no significant difference in antibody production between the mouse GST-EF peptide and GST-only immunization groups. These results indicated that the bacterial EF epitope sequence, QTREHIL, induced a high humoral immune response, and that residue I^115^ is likely to be the key amino acid residue for host recognition of self and non-self protein.

### Anti-epitope EF Peptide Antibodies Exist in *B. melitensis*-Infected Mouse Sera

Serum samples from *B. melitensis*-infected and control mice were examined using synthetic epitope peptide as the antigen (Figure 5E). Dot blot analysis showed that epitope EF reacted with sera from infected mice but not with samples from the uninfected control mice. These data suggested that the EF peptide may be an important antigen epitope in animal hosts.

### Application of the BD_6_ Epitope in a Yeast Expression System

**FIGURE 6:**
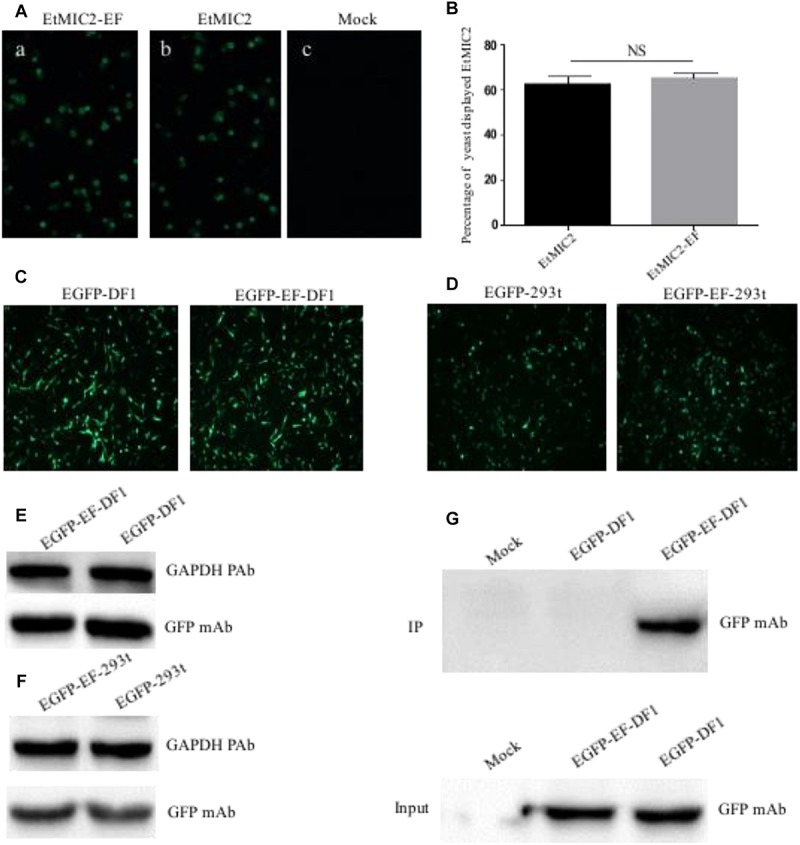
Detection and immunoprecipitation (IP) of EF tag fused protein in yeast, human embryonic kidney cells (HEK-293) and chicken fibroblast cell line (DF1). **(A)** Immunofluorescence analysis of EtMIC2-EF **(a)** and EtMIC2 **(b)** were expressed in yeast cells, using anti-EtMIC2 McAb as primary antibody. Yeast transfected with empty vector as control **(c)**. **(B)** Analyzed quantitative expression of EtMIC2-EF and EtMIC2 on the yeast cells surface by flow cytometry. NS, not significant (*p* > 0.05). **(C)** DF1 cells transfected with pEGFP–C1 and pEGFP–EF showed obvious fluorescence signals at 36 h post-transfection. **(D)** HEK-293 cells transfected with pEGFP–C1 and pEGFP–EF showed obvious fluorescence signals at 36 h post-transfection. **(E,F)** The level of expression in DF1 cell or HEK-293 transfected with pEGFP–C1 and pEGFP–EF was detected by western blot using anti-GFP McAb. GAPDH is loading control. **(G)** IP of analysis of transfected cell lysates using BD6 as a capture antibody in a pull-down assay. Only EGFP-EF was detected by IP using anti-GFP McAb. Mock normal cells.

To examine whether epitope EF-tagged proteins could be successfully expressed in yeast, *S. cerevisiae* cells transformed with recombinant plasmid pCTCON2-EtMIC2-EF or PCTCON2-EtMIC2 were screened using an immunofluorescent labeling assay. Results showed that the green fluorescence was homogeneously distributed on the surface of yeast cells immuno-stained with anti-EtMIC2 McAb (Figure 6A). Flow cytometry showed that 63.1% of yeast cells displayed EtMIC2 on their surface, while 65.3% displayed EtMIC2-EF (Figure 6B). This result suggested that the epitope EF tag did not affect heterogenous gene expression in yeast cells.

### Expression and Immunoprecipitation of the BD_6_ Epitope Tag in Mammalian and Avian Cell Expression Systems

Although the epitope EF-tag was successfully expressed in a yeast expression system, it was unclear whether EF-tagged fusion proteins could be expressed correctly and whether the tag could be of use in protein studies requiring mammalian and avian cell systems. Expression plasmid pEGFP-EF, containing an EF tag fused to the C-terminus of EGFP, was transfected into HEK-293 or DF1 cells and analyzed at 36 h post-transfection. The pEGFP-EF-transfected cells showed obvious green fluorescence signals, similar to those of the positive control (pEGFP-C1) (Figures 6C,D). Western blotting confirmed that there were no differences in expression between pEGFP-EF-transfected and pEGFP-C1-transfected cells (Figures 6E,F). These results indicated that the EF tag did not affect the expression or function of recombinant proteins in either mammalian or avian cell expression systems.

To investigate whether the EF tag and BD_6_ McAb could be used in immunoprecipitation assays, lysates from transfected cells were incubated with BD_6_ McAb, precipitated using protein G Sepharose, and detected using anti-GFP antibodies. Western blotting showed that the BD_6_ McAb captured EGFP-EF specifically, and did not pull down other proteins from pEGFP-C1-transfected or mock-infected cells (Figure 6G). These data therefore confirmed that the BD_6_ McAb and EF tag could be used in mammalian/avian expression systems and were also suitable for use in immunoprecipitation assays for studying protein-protein interactions.

## Discussion

EF-Tu proteins from some pathogenic bacteria play a pivotal role in protein synthesis as well as being novel moonlighting proteins involved in many cellular and disease processes (Marques et al., 1998; Alonso et al., 2002; Sharma et al., 2011; Jiang et al., 2016). Studies have found that membrane-expressed EF-Tu mediates cytoskeletal complex formation (Defeu Soufo et al., 2010; Matsubayashi et al., 2013) and acts as a virulence factor by binding to complement and fibronectin to facilitate infection (Kunert et al., 2007; Balasubramanian et al., 2008). Further studies have shown that EF-Tu can also mediate pathogen invasion (Nandan and Reiner, 2005; Barel et al., 2008) and help evade the host immune response (Matsubayashi et al., 2013). However, few studies have addressed the role of EF-Tu in *Brucella* species, mainly because there is a lack of specific tools for the identification of this protein. McAbs are a useful tool for identifying the function of proteins (Kivi et al., 2016). They can be used in protein subcellular localization studies (Liu et al., 2014), co-immunoprecipitation assays for examining interacting proteins (Verhelst et al., 2015), and for epitope screening (Arthur Huang et al., 2017), amongst other applications. In the present study, we generated a McAb, BD_6_, which specifically recognizes EF-Tu from *B. melitensis*. Using this novel McAb, other functions of the *B. melitensis* EF-Tu protein may be identified, including how it functions during the infection process and its involvement in evading the host immune response.

The epitope (i.e., antigenic determinant) is the pivotal part of an antigen for inducing an immune response. Well-defined epitopes and McAbs provide a platform for studying antigen structure and developing diagnostic methods and epitope vaccines. A recent study showed that the epitope of EF-Tu from *Acidovorax avenae* is the key sequence for inducing an immune reaction in rice (Furukawa et al., 2014). As a multifunction protein, EF-Tu is also an important antigen (Sharma et al., 2011; Matsubayashi et al., 2013; Wang et al., 2013). Following an immunoproteomic analysis of *B. melitensis*, Yang et al. (2011) showed that EF-Tu is a candidate immunogenic protein for developing a brucellosis subunit vaccine. To determine the key amino acid residues of *B. melitensis* EF-Tu, we identified the sequence of a linear B-cell epitope, ^110^QTREHIL^116^, in the *B. melitensis* EF-Tu protein. A dot blot assay confirmed that anti-EF peptide antibodies also exist in serum from *B. melitensis*-infected mice. Accordingly, the epitope EF is likely to be an important B-cell epitope, playing a major role in inducing an immune reaction and with potential for development as an epitope vaccine. Sequence alignment identified only a single amino acid (I^115^) difference between the *B. melitensis* and host EF peptide sequences. Immunization of mice showed that epitope EF can induce a humoral immune response generating highly specific serum antibodies, but that no reaction occurred following immunization with the mouse peptide (QTREHAL). Therefore, residue I^115^ is likely to be a key residue allowing host cells to recognize and differentiate between antigenic and endogenous EF-Tu proteins. This specificity of McAb BD_6_ provides a tool for studying the function and mechanism of *B. melitensis* EF-Tu. Through the application of blastp (BLASTP 2.9.0 +) online analysis on the NCBI website, the results showed that the EF tag is conserved in bacteria species when the parameter Max Target Sequences was set to 100. The epitope peptide was conserved among bacterial species, indicating that this McAb BD_6_ could also be used for identification of other bacterial pathogens.

Affinity tags have been widely used in the purification of recombinant or native proteins and in the exploration of protein function. Together with corresponding McAbs, epitope tags have diverse experimental applications, including western blot analysis, ELISA, co-immunoprecipitation, endogenous localization, and affinity purification (Kimple et al., 2013). Compared with larger affinity tags, such as maltose-binding protein and GST, epitope tags (between 6 and 22 amino acids in length) can minimize effects on the tertiary structures and biological activities of fusion proteins (Terpe, 2003). However, the most commonly used affinity epitope tags have some disadvantages, including sub-optimal affinity or specificity (England et al., 2015) and significant effects on protein expression (Noirclerc-Savoye et al., 2015) and crystallization (Xu et al., 2008). Liu et al. (2016) identified a 12-residue epitope using a McAb that is suitable for applications in bacterial, plant, and mammalian cell systems. However, the epitope is larger than Flag, HA, and Myc tag. In the present study, a 7-residue epitope recognized by BD_6_ was identified by western blotting. Alanine-scanning mutagenesis revealed that BD_6_ specifically recognizes its epitope peptide at five crucial amino acid residues. Indirect immunofluorescence and western blotting assays indicated that epitope EF could be used in yeast, mammalian, and avian expression systems as well as in immunoprecipitation assays for analysis of protein-protein interactions. Based on the small size of the epitope and the high specificity of BD_6_, epitope EF shows great potential for use as a novel epitope tag for exploring protein function.

In conclusion, we generated a McAb, BD_6_, which specifically recognizes bacterial EF-Tu proteins. After identifying the linear B-cell epitope, we examined the central sequence and immunogenicity of the epitope. The resulting sequence, ^110^QTREHIL^116^, is believed to be the first linear epitope identified in the *B. melitensis* EF-Tu protein. This work provides an important epitope for developing epitope vaccines and may be a powerful tool for studying the potential functions of the *B. melitensis* EF-Tu protein. The McAb BD_6_ is a useful tool for further investigation of the potential functions of the EF-Tu protein in pathogen-host interactions, and the epitope tag may be useful for application as a novel affinity tag to identify other bacterial pathogens, which is especially convenient for the identification of intracellular bacteria.

## Data Availability

The raw data supporting the conclusions of this manuscript will be made available by the authors, without undue reservation, to any qualified researcher.

## Author Contributions

FW and XZ participated in the study design. NZ and YJ carried out the study and NZ drafted the manuscript. YJ, SM, and SL collected the important background information. All authors read and approved the final manuscript.

## Conflict of Interest Statement

The authors declare that the research was conducted in the absence of any commercial or financial relationships that could be construed as a potential conflict of interest.
