# Supplementary material for: Monoclonal Antibody Preparation and Epitope Identification for Brucella melitensis Elongation Factor Tu
Source: Front Microbiol. 2019 Aug 13;10:1878. doi: 10.3389/fmicb.2019.01878 (PMC6705223; doi:10.3389/fmicb.2019.01878)
Supplement: Supplementary file 1 [file Table_1.DOCX]

**Table 1. Primers for identification of linear epitope in *B. melitensis* EF-Tu protein.**

| Protein name | Primers | | Sequence (5ʹ-3ʹ) | Size (bp) | AA position |
| --- | --- | --- | --- | --- | --- |
| EF-Tu-1-1 | BM-EF-Tu-F  EF-Tu-1-1-R | TTGGATCCATGGCAAAGAGTAAGTTTGAAC  TTCTCGAGGGTCGGAATGTAGCTGTC | | 597 | 1–199 |
| EF-Tu-1-2 | EF-Tu-1-2-F  EF-Tu-1-2-R | TTGGATCCGTGGTTTCGGCTGCTGAC  TTCTCGAGACCCGGCTTGCAGAGAAC | | 585 | 100–294 |
| EF-Tu-1-3 | EF-Tu-1-3-F  BM-EF-Tu-R | TTGGATCCATCGAAGACGTGTTCTC  TTCTCGAGTTACTCGATGATCGAC | | 543 | 212–392 |
| EF-Tu-2-1 | EF-Tu-2-1-R | TTCTCGAG GATGATCGGGATTTCGTCG | | 201 | 100–166 |
| EF-Tu-2-2 | EF-Tu-2-2-F | TTGGATCC CACATTCTGCTTGCCCGT | | 258 | 114–294 |
| EF-Tu-3-1 | EF-Tu-3-1-F  EF-Tu-3-1-R | GATCCGTGGTTTCGGCTGCTGACGGCCCGATGC  TCGAGCATCGGGCCGTCAGCAGCCGAAACCACG | | 27 | 100–108 |
| EF-Tu-3-2 | EF-Tu-3-2-F  EF-Tu-3-2-R | GATCCTCGGCTGCTGACGGCCCGATGCCGCAGC  TCGAGCTGCGGCATCGGGCCGTCAGCAGCCGAG | | 27 | 102–110 |
| EF-Tu-3-3 | EF-Tu-3-3-F  EF-Tu-3-3-R | GATCCGCTGACGGCCCGATGCCGCAGACCCGCC  TCGAGGCGGGTCTGCGGCATCGGGCCGTCAGCG | | 27 | 104–112 |
| EF-Tu-3-4 | EF-Tu-3-4-F  EF-Tu-3-4-R | GATCCGGCCCGATGCCGCAGACCCGCGAGCACC  TCGAGGTGCTCGCGGGTCTGCGGCATCGGGCCG | | 27 | 106–114 |
| EF-Tu-3-5 | EF-Tu-3-5-F  EF-Tu-3-5-R | GATCCATGCCGCAGACCCGCGAGCACATTCTGC  TCGAGCAGAATGTGCTCGCGGGTCTGCGGCATG | | 27 | 108–116 |
| EF-Tu-4-1 | EF-Tu-4-1-F  EF-Tu-4-1-R | GATCCCCGCAGACCCGCGAGCACATTCTGC  TCGAGCAGAATGTGCTCGCGGGTCTGCGGG | | 24 | 109–116 |
| EF-Tu-4-2 | EF-Tu-4-2-F  EF-Tu-4-2-R | GATCCCAGACCCGCGAGCACATTCTGC  TCGAGCAGAATGTGCTCGCGGGTCTGG | | 21 | 110–116 |
| EF-Tu-4-3 | EF-Tu-4-3-F  EF-Tu-4-3-R | GATCCACCCGCGAGCACATTCTGC  TCGAGCAGAATGTGCTCGCGGGTG | | 18 | 111–116 |
| EF-Tu-4-4 | EF-Tu-4-4-F  EF-Tu-4-4-R | GATCCCAGACCCGCGAGCACATTC  TCGAGAATGTGCTCGCGGGTCTGG | | 18 | 110–115 |

**Table 2. Primers for identification of the key amino acid residue of the epitope to McAb BD_6_**

| Protein name | Primers | Sequence (5ʹ-3ʹ) | | Position |
| --- | --- | --- | --- | --- |
| M- EF-Tu-1 | M- EF-Tu-1 -F  M- EF-Tu-1-R | | GATCCGCCACCCGCGAGCACATTCTGC  TCGAG GTCTTACACGAGCGCCCACCGG | Q^110^-A |
| M- EF-Tu-2 | M- EF-Tu-2-F  M- EF-Tu-2-R | | GATCCCAGGCCCGCGAGCACATTCTGC  TCGAGCAGAATGTGCTCGCGGGCCTGG | T^111^-A |
| M- EF-Tu-3 | M- EF-Tu-3-F  M-EF-Tu-3 -R | | GATCCCAGACCGCCGAGCACATTCTGC  TCGAGCAGAATGTGCTCGGCGGTCTGG | R^112^-A |
| M- EF-Tu-4 | M- EF-Tu-4-F  M- EF-Tu-4-R | | GATCCCAGACCCGCGCGCACATTCTGC  TCGAGCAGAATGTGCGCGCGGGTCTGG | E^113^-A |
| M- EF-Tu-5 | M- EF-Tu-5-F  M- EF-Tu-5-R | | GATCCCAGACCCGCGAGGCCATTCTGC  TCGAGCAGAATGGCCTCGCGGGTCTGG | H^114^-A |
| M- EF-Tu-6 | M- EF-Tu-6-F  M- EF-Tu-6-R | | GATCCCAGACCCGCGAGCACGCACTGC  TCGAGCAGTGCGTGCTCGCGGGTCTGG | I^115^-A |
| M- EF-Tu-7 | M- EF-Tu-7-F  M- EF-Tu-7-R | | GATCCCAGACCCGCGAGCACATTGCGC  TCGAGCGCAATGTGCTCGCGGGTCTGG | L^116^-A |

**Table 3. Primers for mutation of *B. melitensis* EF-Tu protein in the epitope site**

| Protein name | Primers | Sequence (5ʹ-3ʹ) | Position |
| --- | --- | --- | --- |
| P-EF-Tu-(Q^110^-A) | P-EF-Tu-F1 | GTCGACATGGCAAAGAGTAAGTTTGAACGTACG | Q^110^-A |
|  | P-EF-Tu-(110)-R1 | GAATGTGCTCGCGGGTGGCCGGCATCGGGCCGTC |  |
|  | P-EF-Tu-(110)-F2 | GACGGCCCGATGCCGGCCACCCGCGAGCACATTC |  |
|  | P-EF-Tu-R2 | GCTAGCTTACTCGATGATCGACGAGACGATGCC |  |
| P-EF-Tu-(T^111^-A) | P-EF-Tu-(111)-R1 | GCAGAATGTGCTCGCGGGCCTGCGGCATCGGGCCG | T^111^-A |
|  | P-EF-Tu-(111)-F2 | CGGCCCGATGCCGCAGGCCCGCGAGCACATTCTGC |  |
| P-EF-Tu-(R^112^-A) | P-EF-Tu-(112)-R1 | CAAGCAGAATGTGCTCGGCGGTCTGCGGCATCGGG | R^112^-A |
|  | P-EF-Tu-(112)-F2 | CCCGATGCCGCAGACCGCCGAGCACATTCTGCTTG |  |
| P-EF-Tu-(E^113^-A) | P-EF-Tu-(113)-R1 | GGGCAAGCAGAATGTGGGCGCGGGTCTGCGGCATC | E^113^-A |
|  | P-EF-Tu-(113)-F2 | GATGCCGCAGACCCGCGCCCACATTCTGCTTGCCC |  |
| P-EF-Tu-(H^114^-A) | P-EF-Tu-(114)-R1 | GACGGGCAAGCAGAATGGCCTCGCGGGTCTGCGGC | H^114^-A |
|  | P-EF-Tu-(114)-F2 | GCCGCAGACCCGCGAGGCCATTCTGCTTGCCCGTC |  |
| P-EF-Tu-(I^115^-A) | P-EF-Tu-(115)-R1 | CCTGACGGGCAAGCAGGGCGTGCTCGCGGGTCTGC | I^115^-A |
|  | P-EF-Tu-(115)-F2 | CGCAGCCCCGCGAGCACGCCCTGCTTGCCCGTCAGG |  |
| P-EF-Tu-(L^116^-A) | P-EF-Tu-(116)-R1 | CAACCTGACGGGCAAGGGCAATGTGCTCGCGGGTC | L^116^-A |
|  | P-EF-Tu-(116)-F2 | GACCCGCGAGCACATTGCCCTTGCCCGTCAGGTTG |  |

**Table 4. Amino acid sequence of overlapping fragments of the *B. melitensis* EF-Tu**

| Protein name | Amino acid sequence | position |
| --- | --- | --- |
| EF-Tu-1-1 | MAKSKFERTKPHVNIGTIGHVDHGKTSLTAAITKFFGEFKAYDQIDAAPEERARGITISTAHVEYETANRHYAHVDCPGHADYVKNMITGAAQMDGAILVVSAADGPMPQTREHILLARQVGVPAIVVFLNKCDQVDDAELLELVELEVRELLSKYEFPGDEIPIIKGSALAALEDSSKELGEDAIRNLMDAVDSYIPT | 1-199 |
| EF-Tu-1-2 | VVSAADGPMPQTREHILLARQVGVPAIVVFLNKCDQVDDAELLELVELEVRELLSKYEFPGDEIPIIKGSALAALEDSSKELGEDAIRNLMDAVDSYIPTPERPIDQPFLMPIEDVFSISGRGTVVTGRVERGIVKVGEEVEIVGIKATTKTTVTGVEMFRKLLDQGQAGDNIGALIRGVGREDVERGQVLCKPG | 100-294 |
| EF-Tu-1-3 | IEDVFSISGRGTVVTGRVERGIVKVGEEVEIVGIKATTKTTVTGVEMFRKLLDQGQAGDNIGALIRGVGREDVERGQVLCKPGSVKPHTKFKAEAYILTKDEGGRHTPFFTNYRPQFYFRTTDVTGVVTLPAGTEMVMPGDNVAMDVTLIVPIAMEEKLRFAIREGGRTVGAGIVSSIIE | 212-392 |
| EF-Tu-2-1 | VVSAADGPMPQTREHILLARQVGVPAIVVFLNKCDQVDDAELLELVELEVRELLSKYEFPGDEIPII | 100-166 |
| EF-Tu-2-2 | HILLARQVGVPAIVVFLNKCDQVDDAELLELVELEVRELLSKYEFPGDEIPIIKGSALAALEDSSKELGEDAIRNLMDAVDSYIPT | 114-294 |
| EF-Tu-3-1 | VVSAADGPM | 100-108 |
| EF-Tu-3-2 | SAADGPMPQ | 102-110 |
| EF-Tu-3-3 | ADGPMPQTR | 104-112 |
| EF-Tu-3-4 | GPMPQTREH | 106-114 |
| EF-Tu-3-5 | MPQTREHIL | 108-116 |
| EF-Tu-4-1 | PQTREHIL | 109-116 |
| EF-Tu-4-2 | QTREHIL | 110-116 |
| EF-Tu-4-3 | TREHIL | 111-116 |
| EF-Tu-4-4 | QTREHI | 110-115 |
